# HPV prevalence in the foreskins of asymptomatic healthy infants and children: Systematic review and meta-analysis

**DOI:** 10.1038/s41598-017-07506-z

**Published:** 2017-08-01

**Authors:** Bora Lee, Sang Wook Lee, Dae In Kim, Jae Heon Kim

**Affiliations:** 1 Department of Biostatistics, Clinical Trial Center, Soonchunhyang University Bucheon Hospital, Bucheon, Korea; 2 Department of Statistics, Graduate School of Chung-Ang University, Seoul, Korea; 3 Department of Urology, Soonchunhyang University Bucheon Hospital, Soonchuhyang University Medical College, Bucheon, Korea; 4 Department of Pharmaceutical engineering, Soonchunhyang University, Asan, Korea; 5 Department of Urology, Soonchunhyang University Hospital, Soonchuhyang University Medical College, Seoul, Korea

## Abstract

The true HPV prevalence in the foreskins of infants and children has been little documented, but reporting on this prevalence is of great importance given its impact on the rationale for treating asymptomatic boys. We searched multiple databases from 1960 to 2016 for observational or prospective studies that reported on HPV prevalence in foreskins. We conducted a meta-analysis using a random-effects model to pool for HPV prevalence in the foreskins of infants and children. Eight studies, with a total of 556 infants and children with phimosis, were eligible for the meta-analysis. The pooled overall prevalence of general HPV, high-risk HPV, low-risk HPV, HPV 16/18, HPV 16, and HPV 18 were 17.3 (95%CI: 0.8–46.3), 12.1 (95% CI: 0.9–31.5), 2.4 (95% CI: 0.0–11.2), 4.8 (95% CI: 0.0–16.8), 1.7 (95% CI: 0.0–5.1), and 0 (95% CI: 0–0.5), respectively. The estimated HPV prevalence in foreskins was not zero among infants and children, which implies HPV transmission other than by sexual contact. Considering that high-risk HPV is detected in asymptomatic infants and children, future studies are warranted to determine whether preventive treatments in asymptomatic infants and children could be effective in preventing persistence or transmission of high-risk HPV.

## Introduction

Human papillomavirus (HPV) infection is a threat to public health, causing medical problems from warts to cancer including cervical and penile cancer^1^. Among these, cervical cancer is a great burden and a source of significant fatality. Nearly all, 99%, cervical cancer cases involve malignant genetic types of HPV^2^. In recent decades, studies on HPV have been confined to sexually transmitted HPV and can be categorized into one of two topics: investigating the detailed mechanism of malignant HPV in inducing or aggravating cervical and penile cancer, and the potential role of preventive treatment including circumcision and vaccination.

Circumcision is a relatively simple procedure without severe complications, and it is recommended because of its benefits in preventing sexually transmitted disease including human immunodeficiency virus (HIV) and HPV^3, 4^. However, there still remains great controversy on performing circumcision to prevent genital infection, which could help to prevent cervical and penile cancer. There are several systematic meta-analyses on this topic, but they present different outcomes. The two most recent meta-analyses, by Van Howe *et al*.^5^ and Zhu *et al*.^6^, indicate a limited role of circumcision in preventing genital HPV infections; the authors concluded that there is no association between circumcision and protection against HPV infection because even reduced HPV prevalence by circumcision does not reflect reduced HPV acquisition and clearance.

The reasons for the controversy about this issue lie in the nature of the studies conducted, including few randomized clinical trials (RCTs), selection bias, reporting bias related to sampling, and measurements. Clinicians have never focused on the fact that all these studies have had selection bias in that most have included males with a sexual contact history. The preventive role of circumcision for HPV infection could compromise or overestimate the effects of circumcision because of possible preexisting HPV or pre-malignant lesions. Moreover, there are issues of sample accuracy unless specimens are acquired by circumcision because the foreskin has great importance in carrying HPV.

To demonstrate this issue, RCTs are necessary with samples of infants or children with extremely long follow-up; however, such studies would be both costly and difficult to execute. Before such RCTs are performed, and also to discuss the role of circumcision in preventing genital HPV infection related to cervical and penile cancer, the first step would be to investigate the true HPV prevalence among males without a sexual contact history. Although clinicians seldom focus on non-sexual routes of HPV transmission, there is evidence of other modes of transmission including vertical and horizontal^7, 8^.

The aim of this study was to investigate the real prevalence of HPV in foreskin samples from males without previous sexual contact. This investigation could remind clinicians of the importance of circumcision in HPV transmission.

## Methods

To report our prevalence data, we performed a systematic review and meta-analysis without language restrictions in accordance with the Preferred Reporting Items for Systematic Reviews and Meta-Analyses statement^9^ and with previous experience with meta-analysis^10^.

### Types of studies and participants

We included both observational and prospective clinical studies that addressed foreskins and HPV prevalence. Our sole inclusion criterion was being an infant or child on the assumption that very young children would not have had any sexual contact. Moreover, to obtain normal samples, we excluded penile inflammation. We collected the HPV DNA from foreskin tissue samples.

### Search methods for identifying studies and outcome measures

We conducted a cross-search of all related literature in MEDLINE through June 2016 and used an optimally sensitive Cochrane Collaboration search strategy using MeSH headings including “papillomavirus infections,” “papillomaviridae,” “foreskin,” and “circumcision.” For natural language headings, we included “papilloma virus infection,” “papilloma virus infections,” “papilloma virus,” “papilloma viruses,” “papillomavirus,” “papillomaviridae,” “wart virus,” “HPV,” “human papilloma virus,” “human papilloma viruses,” “human papillomavirus,” “foreskin,” “prepuce,” and “circumcision.” We also searched EMBASE from 1980 to June 2016 and the Cochrane Library.

We included studies if they met the following criteria: (i) HPV prevalence determined in infants and children without a history of sexual contact; (ii) specimens had been obtained from foreskins; and (iii) the infants and children had asymptomatic foreskins. From each study, we recorded data on first author, publication year, study location, sample size, participant age range, and investigated HPV type. We extracted the data on HPV prevalence for general HPV (any type), high-risk HPV (HR-HPV), low-risk HPV, HPV 16, HPV 18, and HPV 16/18 (HPV 16 or HPV 18).

### Data collection and analysis

The initial screening of the electronic databases for study selection was based on information in the title and abstract. Two independent authors (D.I. Kim and J.H. Kim) conducted the screening; we screened the studies and reviewed the complete study reports for selection. In cases of insufficient data, the authors reviewed the full text of the article for further information which was either reported directly or reported that could be converted to the required values. All authors discussed the studies before final selection. The authors carefully cross-checked the references and data for each included study to ensure that no overlapping data were present and to maintain the integrity of the meta-analysis.

### Statistical analyses

For prevalence of type-specific HPV, because we only included studies that tested for a particular HPV type in our analyses of that type, sample size varied by HPV type. To calculate the pooled prevalence of HPV, we first transformed the prevalence from each study using Freeman-Tukey double arcsine transformation^11^; arcsine transformations contribute to stabilizing the variance of simple proportions. We calculated the pooled prevalence as the back-transformation of the weighted means of the transformed prevalences, using Dersimonian-Laird weights for the random-effects model. The reason we used a random-effects model was the expected heterogeneity in the study for locations, populations sampled, and HPV types investigated.

We conducted meta-regression analysis for HPV prevalence to examine the potential moderators. We analyzed the variability in the effect sizes due to differences between the moderators (e.g., location of studies, median age, and publication year) with a restricted maximum likelihood estimator of the variance of the true effects.

We assessed heterogeneity across studies using *P* with the Q statistic and the I^2^ statistic, categorized as follows: <30% not important; 30–50% moderate; 50–75% substantial; and >75% considerable^12^. We used Galbraith plots to spot the outliers and conducted sensitivity analyses by examining the effects of excluding those outliers. To assess the effects of individual studies on the pooled estimates, we conducted influential analysis by omitting each study and re-estimating.

When we were synthesizing the effect sizes of each study, we eliminated the outlier data to obtain more valid effect sizes. First, we examined the presumed effect sizes of any extreme data to determine whether they were outliers; then we judged whether to include or exclude the data during synthesis. We performed all analyses using R software version 3.1.3 (The R Foundation for Statistical Computing, Vienna, Austria).

### Assessing the risk of bias in the included studies

Two reviewers independently assessed the methodological quality of the studies and the data extraction, and discrepancies were resolved by consensus. We assessed risk of bias using the Quality in Prognosis Studies (QUIPS) tool^13^.

### Assessing for reporting biases

We conducted meta-analyses for small study effects using Begg and Mazumdar’s rank correlation test and Egger’s linear regression method test for publication biases. In particular, we show separately the *P*-values for the two statistical tests for all antibiotic types.

## Results

### Inclusion of studies

**Figure 1:**
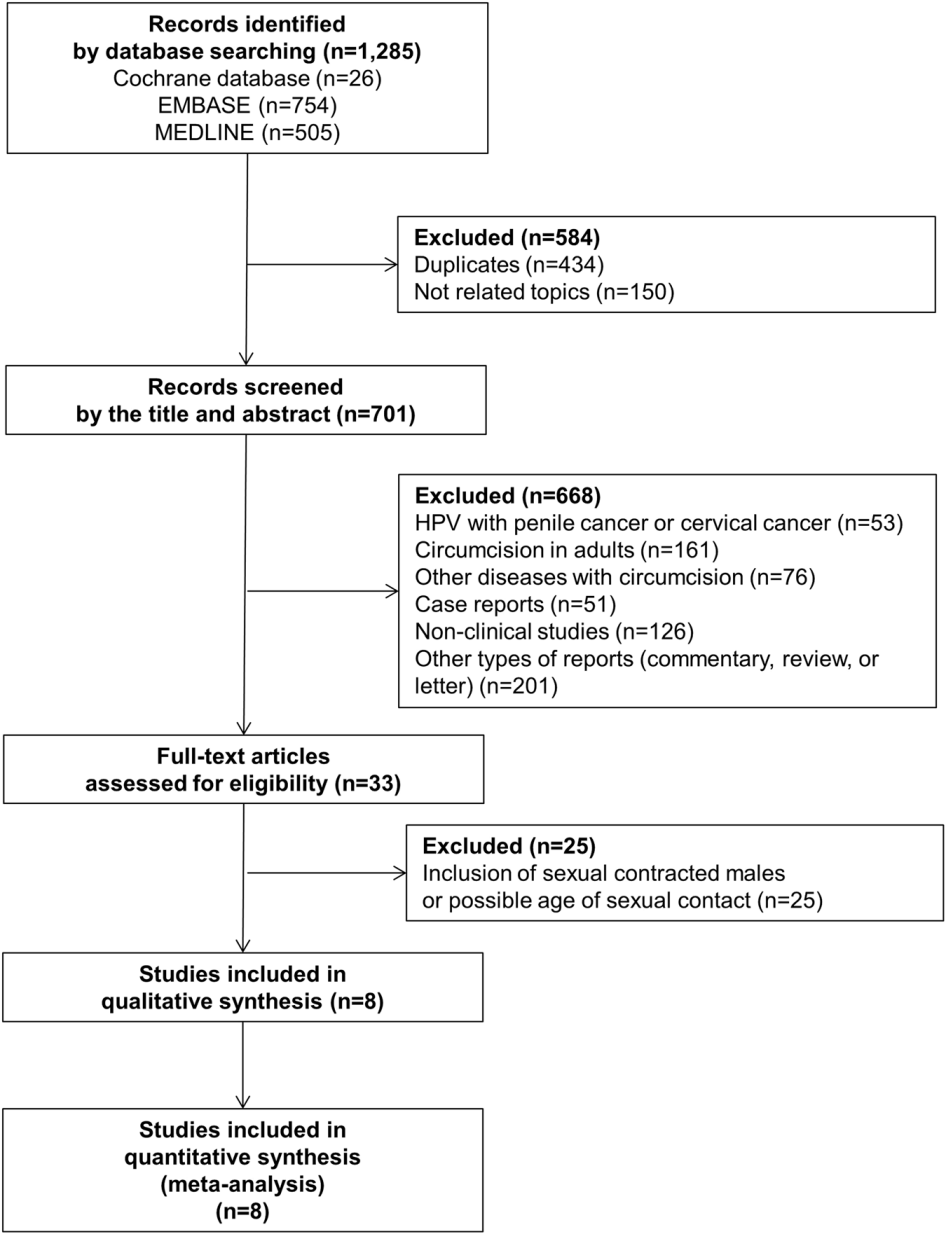
Flow chart of the included studies.

The initial search identified a total of 1285 articles from electronic databases (MEDLINE, 505; Cochrane, 26; EMBASE, 754). After we eliminated 434 studies that contained overlapping data or that appeared in more than one database and after we screened the titles and abstracts, we identified 701 studies as eligible for intensive screening. Of these, we eliminated 53 studies relating to penile or cervical cancer; 161 on circumcision and HPV clinical studies in adults; 76 on other diseases with circumcision; 51 case reports; 126 non-clinical studies; and 201 other types of studies including commentary, reviews, and letters. A total of 33 studies fit the selection criteria, but 25 of them included cases with possible sexual contact. Finally, we used 8 studies that met all the inclusion criteria. These 8 studies comprised a total of 556 subjects for HPV prevalence (556 for prevalence of general HPV, HR-HPV, LR-HPV, and HPV 16/18 and 405 for prevalence of HPV 16 and HPV 18). A detailed flow chart showing the selection process is shown in Fig. 1. The included 8 studies^14–21^ contained detailed research duration and subject description information (Table 1). The research durations ranged from 1986 to 2015.

**Table 1:** Characteristics and results of the included studies.

Author (year)	Location	No. of samples	Median age (Range)	Phimosis	Investigated HPV type	general HPV	HR-HPV	LR-HPV	HPV type		
									16/18	16	18
Balci *et al*.^15^	Turkey	100	5.7 (0.2–9.0)	55	16, 18, 31, 33, 35, 39, 45, 51, 52, 56, 58, 59, 66, 68	9	9	0	3	3	0
Maarof *et al*.^14^	Malaysia	51	9.0 (4.0–12.0)	2	NA	2	0	2	0	0	0
Klinglmair *et al*.^18^	Austria	121	NA (0.0–10.0)	NA	6, 11, 16, 18	98	55	43	55	NA	NA
Pilatz *et al*.^16^	Germany	82	4.1 (1.0–14.0)	82	6, 11, 16, 18, 31, 33, 35, 39, 42, 44, 45, 51–54, 56, 58, 59, 61, 62, 66–68, 70, 72, 73, 81–84, 90, 91	0	0	0	0	0	0
Martino *et al*.^17^	Austria	50	5.5 (0.42–15)	NA	16, 18, 31, 33, 35, 39, 45, 51, 52, 56, 58, 59	6	6	0	6	6	0
Verit *et al*.^21^	Turkey	30	8.1^a^ (4.0–11.0)	NA	16, 18, 31, 33, 35, 39, 45, 51, 52, 56, 58, 59, 66, 68	25	25	0	1	NA	NA
Chen *et al*.^19^	Austria	52	Neonates^b^	NA	NA	0	0	0	0	0	0
Roman *et al*.^20^	U.S.	70	Neonates^c^	NA	6, 11, 16, 18	3	2	3	2	2	0

NA, not available; LR, low-risk; HR, high-risk.

^a^Mean; ^b^Sampled within hours of birth; ^c^Sampled within 3 days of birth.

### Quality assessment and reporting bias

Table 2 shows the quality assessment of the included studies using the QUIPS tool. All of the study authors reported the detailed reasons for their selected populations and included detailed descriptions of the sampling and measurement methods. Study participation bias was low in all studies except for one^16^ in which the authors included normal foreskins but some subjects had foreskin problems. Outcome measurement bias was low in all studies except for one^14^ in which the authors did not provide a detailed description of how they measured outcomes.

**Table 2:** Risk of bias for included studies.

Author (year)	Location	Detailed reasons for selected population	Detailed description of sampling and measurement method	Risk of bias	
				study participation	outcome measurement
Balci *et al*.^15^	Turkey	The reasons for circumcision were primary phimosis and religious.	Yes	Low	Low
Maarof *et al*.^14^	Malaysia	Target cohort was used	No	Low	Unclear
Klinglmair *et al*.^18^	Austria	Male individuals (without HPV related lesions) after circumcision due to congenital (children, adolescents).	Yes	Low	Low
Pilatz *et al*.^16^	Germany	The boys were referred for urological consultation due to foreskin problems.	Yes	High	Low
Martino *et al*.^17^	Austria	All boys referred to the pediatric urology unit of our department for radical circumcision of primary phimosis and did not show any signs or symptoms suggestive of HPV infection.	Yes	Low	Low
Verit *et al*.^21^	Turkey	The reasons for circumcision were mostly religious, with hypospadias repair in 3 patients.	Yes	Low	Low
Chen *et al*.^19^	Austria	Consecutive neonates who underwent routine autopsy.	Yes	Low	Low
Roman *et al*.^20^	U.S.	Unselected infants undergoing routine circumcision	Yes	Low	Low

### Outcome and findings for HPV prevalence

**Figure 2:**
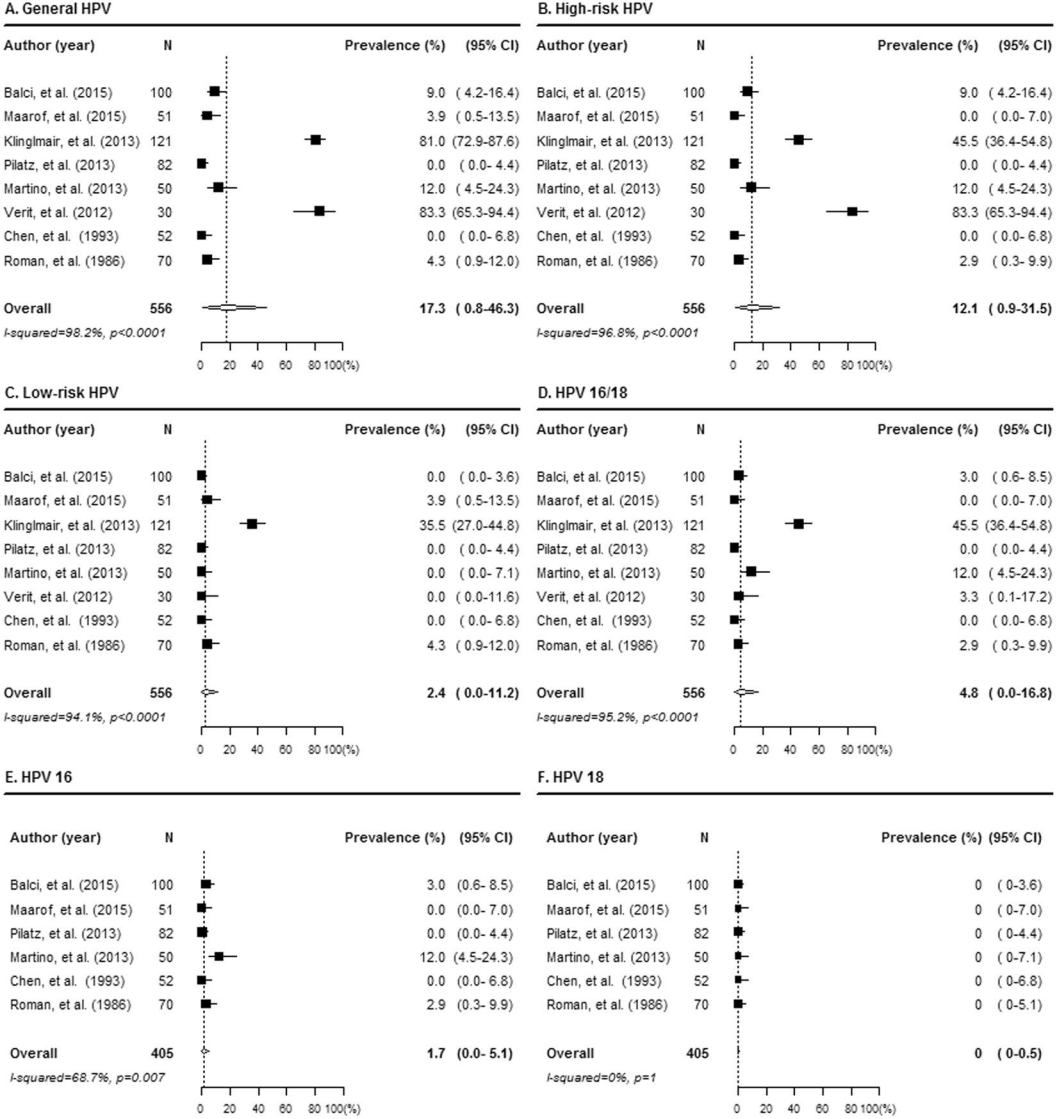
Prevalence of PCR-detected HPV infection. Forest plot diagram showing the pooled estimates for HPV prevalence for general HPV (**A**), high-risk HPV (**B**), low-risk HPV (**C**), HPV 16/18 (**D**), HPV 16 (**E**), and HPV 18 (**F**). The black square signifies the weighted mean of each estimate. All data provided are for continuous outcomes.

Detailed findings for HPV prevalence are described in Table 3 and Fig. 2. The pooled overall prevalence of general HPV, high-risk HPV, low-risk HPV, HPV 16/18, HPV 16, and HPV 18 were 17.3 (95% confidence interval [CI]: 0.8–46.3), 12.1 (95% CI: 0.9–31.5), 2.4 (95% CI: 0.0–11.2), 4.8 (95% CI: 0.0–16.8), 1.7 (95% CI: 0.0–5.1) and 0 (95% CI: 0–0.5), respectively (Table 3 and Fig. 2).

**Table 3:** Estimated prevalence by the random-effect model.

Author (year)	No. of samples	Prevalence (95% CI)^a^					
		general HPV	HR-HPV	LR-HPV	HPV16/18	HPV16	HPV18
Balci *et al*.^15^	100	9.0 (4.2–16.4)	9.0 (4.2–16.4)	0.0 (0.0–3.6)	3.0 (0.6–8.5)	3.0 (0.6–8.5)	0.0 (0.0–3.6)
Maarof *et al*.^14^	51	3.9 (0.5–13.5)	0.0 (0.0–7.0)	3.9 (0.5–13.5)	0.0 (0.0–7.0)	0.0 (0.0–7)	0.0 (0.0–7.0)
Klinglmair *et al*.^18^	121	81.0 (72.9–87.5)	45.5 (36.4–54.8)	35.5 (27.1–44.8)	45.5 (36.4–54.8)	NA	NA
Pilatz *et al*.^16^	82	0.0 (0.0–4.4)	0.0 (0.0–4.4)	0.0 (0.0–4.4)	0.0 (0.0–4.4)	0.0 (0.0–4.4)	0.0 (0.0–4.4)
Martino *et al*.^17^	50	12.0 (4.5–24.3)	12.0 (4.5–24.3)	0.0 (0.0–7.1)	12.0 (4.5–24.3)	12.0 (4.5–24.3)	0.0 (0.0–7.1)
Verit *et al*.^21^	30	83.3 (65.3–94.4)	83.3 (65.3–94.4) NA^†^	0.0 (0.0–11.6)	3.3 (0.1–17.2)	NA	NA
Chen *et al*.^19^	52	0.0 (0.0–6.9)	0.0 (0.0–6.9)	0.0 (0.0–6.9)	0.0 (0.0–6.9)	0.0 (0.0–6.9)	0.0 (0.0–6.9)
Roman *et al*.^20^	70	4.3 (0.9–12.0)	2.9 (0.4–9.9)	4.3 (0.9–12)	2.9 (0.4–9.9)	2.9 (0.4–9.9)	0.0 (0.0–5.1)
Overall	556	17.3 (0.8–46.3)	12.1 (0.9–31.5) 5.9 (0.0–19.8)	2.4 (0.0–11.2)	4.8 (0.0–16.8)	1.7 (0–5.1)	0.0 (0.0–0.5)
Heterogeneity - I^2^ (%)		98.2 (97.5–98.7)	96.8 (95.3–97.8) 95.7 (93.2–97.3)^†^	94.1 (90.5–96.3)	95.2 (92.5–96.9)	68.7 (26–86.7)	0.0 (0.0–0.0)
p-value		<0.001	<0.001 < 0.001^†^	< 0.001	< 0.001	0.007	1

NA, not available; LR, low-risk; HR, high-risk; CI, confidence interval.

^†^This value is generated by omitting the study of Verit *et al*., which was proved to be outlier by Galbraith plot.

^a^The process of meta-analysis with prevalence data: 1) transform the prevalence into a quantity (Freeman-Tukey variant of the arcsine square root transformed proportion), 2) calculate the pooled prevalence as the back-transformation of the weighted mean of the transformed prevalences using DerSimonian-Laird weights assuming the random-effect model.

**Figure 3:**
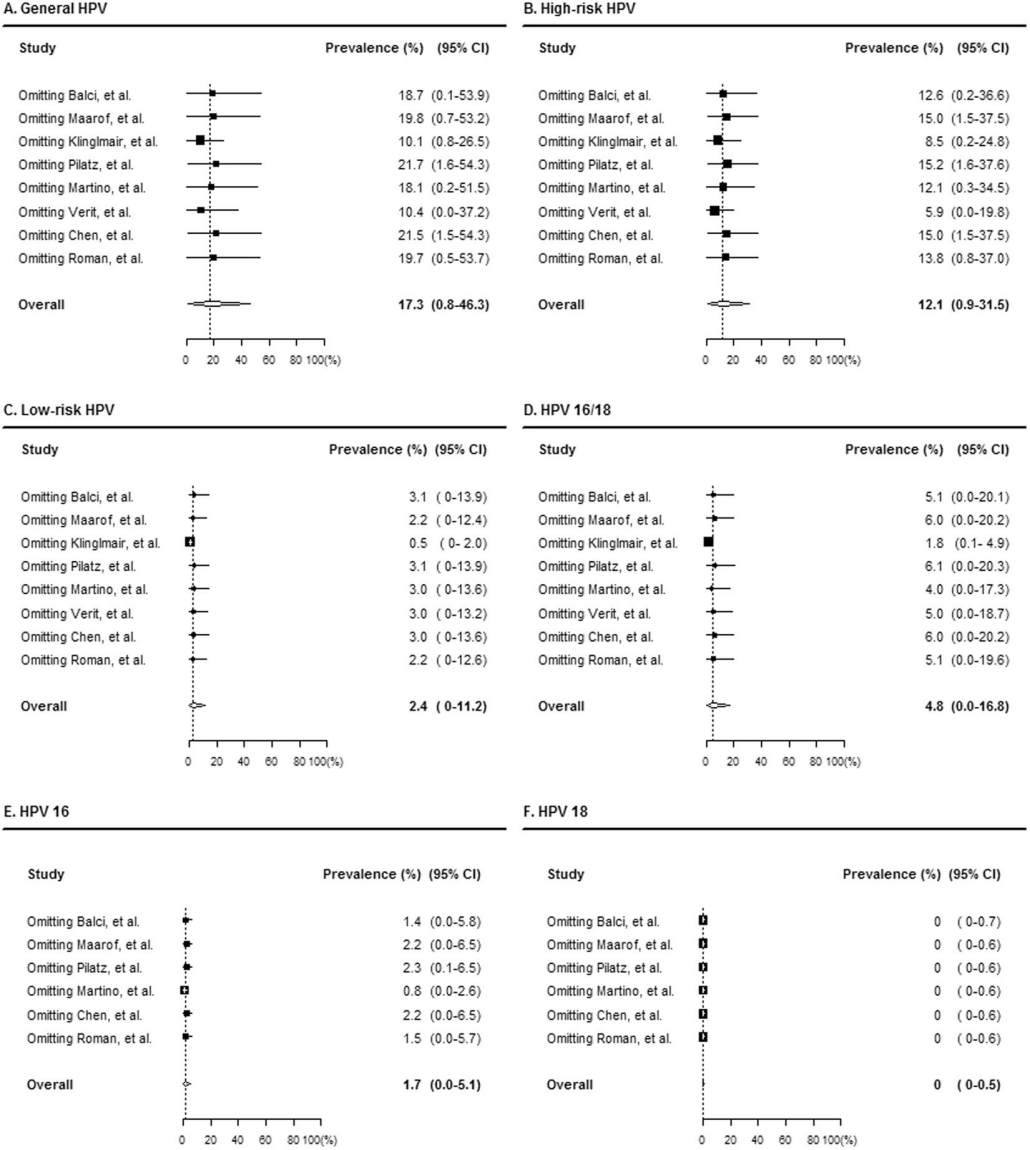
Influential analysis of the prevalence of PCR-detected HPV infection. Forest plot diagram showing the pooled estimate for HPV prevalence after the exclusion of the relevant studies for each type on general HPV (**A**), high-risk HPV (**B**), low-risk HPV (**C**), HPV 16/18 (**D**), HPV 16 (**E**), and HPV 18 (**F**). The black square signifies the weighted mean of each estimate. All data provided are for continuous outcomes.

**Figure 4:**
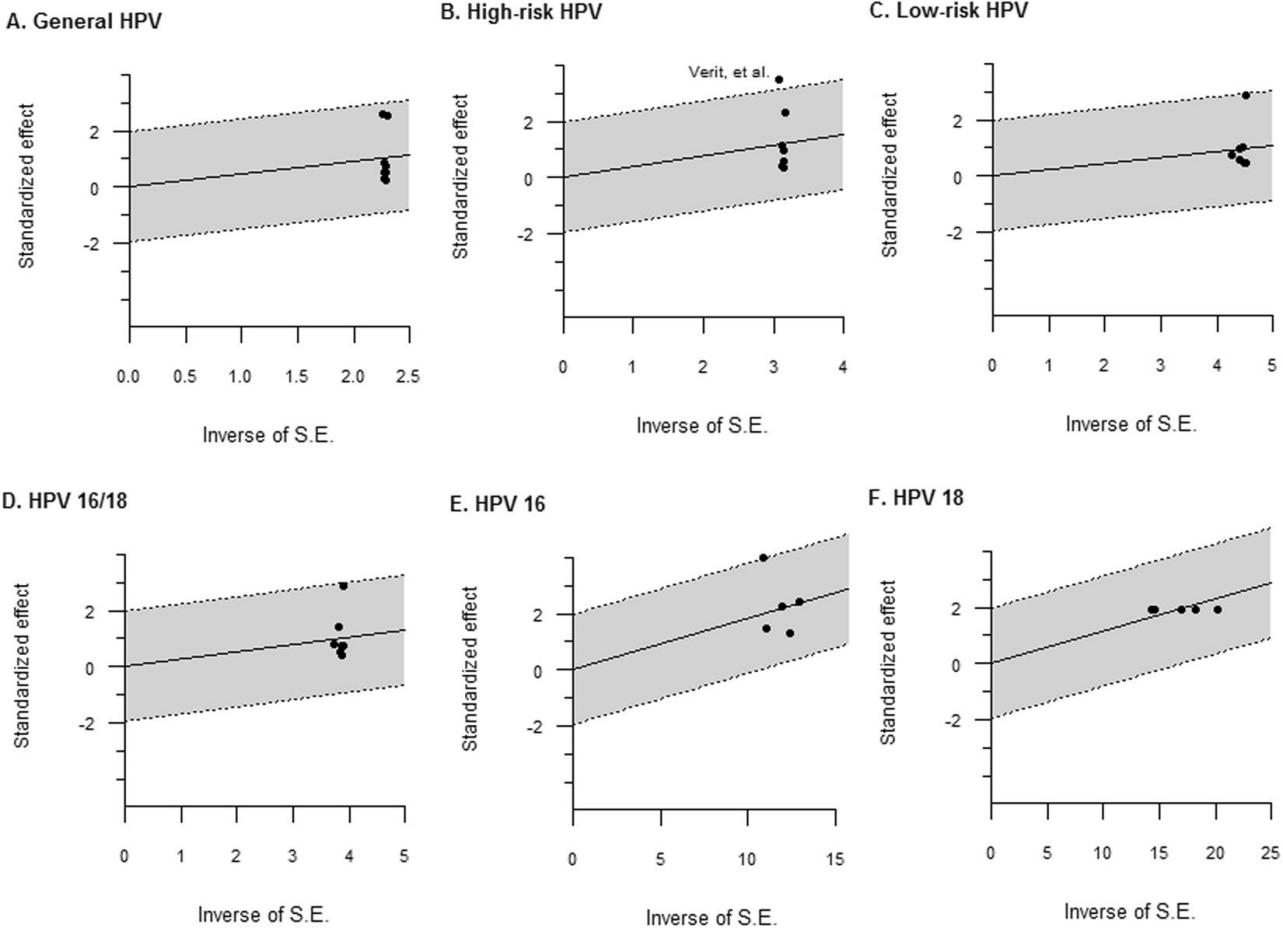
Galbraith plot to spot outliers for estimated meta-prevalence of HPV.

To control for heterogeneity and also to investigate the residual influential effects of each study, we conducted sensitivity analyses (Fig. 3), and all of the HPV prevalence data had been influenced by two studies^18, 21^. Although the study by Verit *et al*.^21^. had a marginal risk of being an outlier, Fig. 3 does not reveal definite outliers for these two studies. However, because Fig. 4 reveals how we determined the outliers for Verit *et al*.^21^ when we analyzed HR-HPV prevalence, we described overall HR-HPV prevalence differently in Table 3 as 5.9 (95% CI: 0.0–19.8).

To determine the potential moderating factors for HPV prevalence, we conducted meta-regression (Table 4) and established that possible moderators including study location, median age, and publication year did not affect the estimated outcomes for HPV prevalence.

**Table 4:** Meta-regression analysis for the prevalence of HPV.

Prevalence (dependent variable)	Location		Median age		Publication year	
	R^2^ (%)^a^	p-value^b^	R^2^ (%)^a^	p-value^b^	R^2^ (%)^a^	p-value^b^
general HPV	0.00	0.606	0.00	0.267	0.00	0.399
HR-HPV	0.00	0.426	0.23	0.204	0.00	0.378
LR-HPV	0.00	0.611	0.00	0.934	0.00	0.913
HPV 16/18	0.00	0.541	0.00	0.826	0.00	0.622
HPV 16	0.00	0.758	0.00	0.888	0.00	0.864
HPV 18	NA		NA		NA	

NA, not available.

^a^The amount of heterogeneity accounted for by the moderator.

^b^p-value for the Wald-type test of the moderator.

### Publication bias

**Figure 5:**
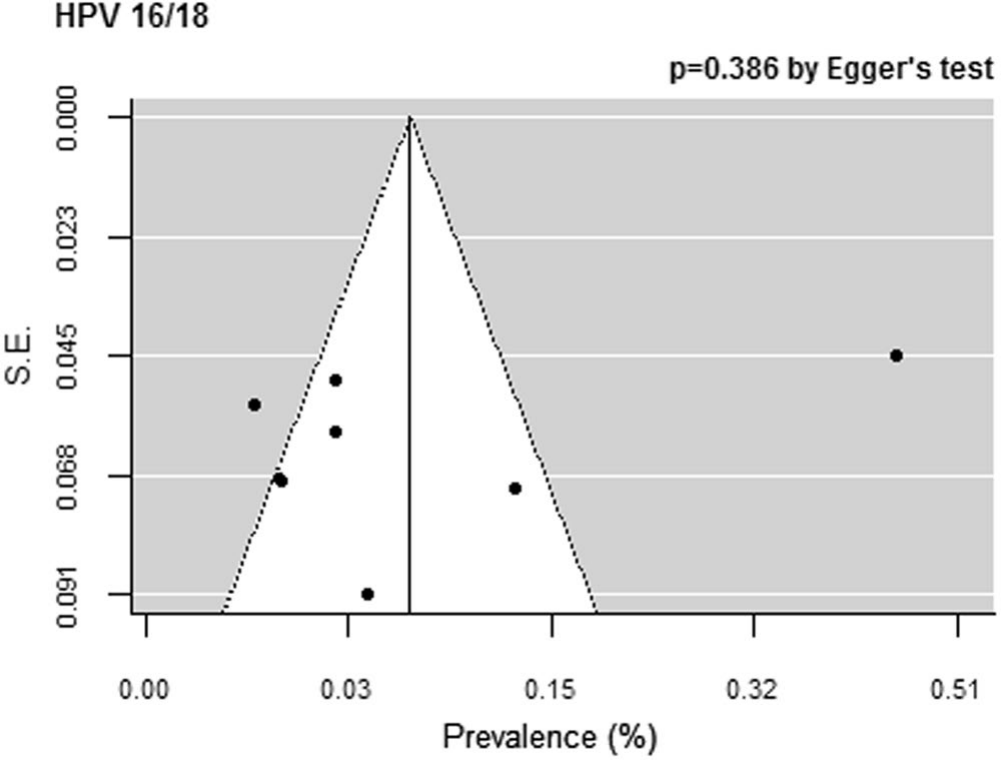
Publication bias for the prevalence of PCR-detected HPV infection. Funnel plots for HPV 16/18. The black square signifies the weighted mean of each estimate. All data provided are for continuous outcomes.

The publication bias findings for all HPV types are shown in Fig. 5. Although we could not show symmetry owing to the large degree of heterogeneity, Egger’s test showed no significance for all HPV prevalence.

## Discussion

The current systematic review with meta-analysis regarding the issue of circumcision and HPV features two salient points: first, we only included subjects who had not had sexual contact, and second, the investigated anatomical target was foreskins. A recent study supports our analyses by showing that HPV 6 and 11, the most common genotypes, can be transmitted earlier in life and persist for long periods including through adolescence, which is the most common period of sexual contact^22^. This phenomenon warrants circumcision or HPV vaccination before a boy reaches adolescence. Although this study does not show direct evidence of the preventive role of circumcision or HPV vaccination before adolescence, our estimated findings for non-zero HPV prevalence in infants and children could reflect two main lessons: the possibility of non-sexual HPV transmission routes and the importance of HPV prevalence in asymptomatic infants and children. Both of these warrant future studies on the preventive role of circumcision or HPV vaccination and the long-term prevalence of HPV in those ages.

In our study, by estimating non-zero HPV prevalence in asymptomatic infants and children, it is clear that there are transmission routes other than sexual contact. To date, this is the first meta-analysis to analyze quantitative and qualitative data on HPV prevalence. Despite having limited data and heterogeneity, we provided firm academic arguments for the need for preventive treatment including circumcision and vaccination in asymptomatic infants and children with no sexual contact history.

There are still controversies^8, 23^ on the transmission routes in infants and children, but prevalence alone is important to consider given the abovementioned persistence of HPV. Although HPV infection could be temporary and incidental, persistent infections are reported as being 10% for oral lesions and 2% for genital lesions. For transmission routes, there are three main categories to consider, vertical and horizontal transmission and autoinoculation. A recent meta-analysis showed that vertical transmission accounts for approximately 20% of total HPV cases^22^. Hence, by analogy, the assumed prevalence of persistent genital HPV infection is 0.4% for total HPV infections. Considering the high prevalence of HIV infections and cervical cancer, 0.4% is not a small number.

Vertical transmission has three components: first, transmission during fertilization; second, prenatal transmission with HPV being detectable in the amniotic fluid, fetal components, cord, and placenta^23^; and third, perinatal transmission by direct contact with HPV during birth through the female genital organs^22, 24^. Horizontal transmission includes skin-to-skin transmission. The prevalence of skin HPV infection is relatively high for children older than 5 years, when they go to school for the first time^25^. Another horizontal transmission mode is nosocomial infection by medical instruments^7^. Although we did not demonstrate systematic results for specific transmission routes, all the included studies had strict inclusion of non-sexual routes of transmission.

The second salient point of this study was that we investigated the HPV prevalence in foreskins. The inconsistent results of many clinical studies and meta-analyses regarding the beneficial role of circumcision can be attributed to various reporting heterogeneities within the studies themselves. These heterogeneities could have been from using inconsistent definitions of HPV infection, differences in the samples used (from biopsy, cotton swabs, or circumcision samples), the HPV DNA detection assay used, and whether or not sampled lesions were used^22^. Among these factors, use of sampled lesions is of great importance because only foreskin samples are related to circumcision. HPV prevalence in oral or anal lesions is not particularly high, and the possibility of obtaining false rates is high for samples from those areas; however, the readings for HPV prevalence in foreskins could be more accurate given the persistent characteristics of HPV detected in foreskins^8^. As described in Table 3, we calculated the overall prevalence of HR-HPV using two estimates: with or without considering the outlier studies.

The anatomical features of the foreskin as a positive reservoir of HPV include redundant skin layers and also moisture, which could result in skin scratches or micro-trauma and infection by other organisms. Usually, HPV itself cannot penetrate the skin epithelium, but with trauma, HPV can reach skin basal cells^7^. Moreover, the mucosa of the foreskin does not contain squamous epithelial keratinization, covering the penis shaft and protecting it from micro-injuries^26^.

The inconsistent results for the protective effects of circumcision also lie in the reporting bias of HPV prevalence, with many studies having reported on HPV prevalence not only in the foreskin but also in other sites including the coronal sulcus, penis shaft, and urethra^1^. Moreover, HPV prevalence studies exist that do not include circumcision, and the value of sampling from other sites including the penis shaft and scrotum is controversial. However, there is certainty in the value of using foreskin samples. On this issue, Castellsague *et al*. reported that male circumcision had a protective effect for cervical cancer risk in female sexual partners without considering the penis shaft and scrotum^4^. HPV in the penis shaft or scrotum could be clinically irrelevant to sexual transmission^22^.

## Limitations

Although this study has attributes including non-sexual origin samples for HPV from infants and children and investigating HPV prevalence in foreskins from circumcision specimens, there were still several limitations. First, we only included a small number of studies that had small sample sizes, and limited information including specific proportions of study samples could affect standard estimates, although the impact is greater for sample size. In this meta-analysis setting, insufficient sample sizes are the main reason for possible increased standard estimates. Second, our study reports on the variability of HPV prevalence. Although we adjusted for the two main issues by including infants and children who had had no sexual contact and by using foreskins as the sampled tissue, there were too few included studies to represent a worldwide pattern of HPV prevalence. In addition, most studies were from only certain countries, and although we primarily included studies that used random sampling, the wide range in the subjects’ ages is another problem. Lastly, we could not predict the long-term beneficial effects of circumcision for HPV infection, which was the fundamental goal of this study. However, only a few studies have researched the long-term effects of circumcision among randomly sampled subjects with HPV that was not transmitted sexually^7, 27^. Moreover, the current detection technique has the intrinsic limitation of assaying for HPV DNA but not discriminating for transient infections or contamination from true HPV infections^22^.

The striking features of researching non-sexually contracted HPV infection include the ability to determine a true prevalence using foreskins from infants and children and the persistence of HPV infection in foreskins. Although we could not conclusively determine the prevalence of non-sexually contracted HPV infection, we did conduct pioneer work in this area. For the issue of persistent HPV, additional studies are required. In particular, longitudinal studies are needed to confirm the role of circumcision and also ascertain the natural history of non-sexually contracted HPV infection in infants and children.

## Conclusions

In this study, for the first time, we revealed the general HPV prevalence and HR-HPV prevalence of non-sexually contracted HPV in infants and children; we demonstrated the existence of non-sexual transmission routes including vertical and horizontal transmission. Although there is heterogeneity in gauging HR-HPV prevalence, considering the persistence of HPV in foreskins, circumcision could still be a valuable option.
